# A novel multi-layered immune structural model for peripheral blood immune scoring in cancer patients: perspective and hypothesis

**DOI:** 10.3389/fimmu.2025.1675411

**Published:** 2025-10-29

**Authors:** Hao Jin

**Affiliations:** Clinical Research Management Department, Tianjin Cancer Hospital Airport Hospital, Tianjin, China

**Keywords:** peripheral blood immune profiling, immune structural model, immune scoring system, tumor immune microenvironment, immunotherapy biomarkers

## Abstract

Accurately assessing and quantifying immune competence in cancer patients remains a major challenge in tumor immunology. Traditional intratumoral immune profiling, such as tissue pathology and tissue-based cytometry techniques, faces significant challenges due to difficulties in tissue sampling, spatial heterogeneity, and technical limitations. In contrast, peripheral blood immune profiling is a more practical and reproducible approach, providing valuable insights into systemic immune status. This article introduces a novel immune structural model, inspired by protein structural hierarchy, to classify immune components into three hierarchical levels: primary, secondary, and tertiary immune structures. We hypothesize that this model can provide a systematic framework for constructing an immune scoring system (ISS) that integrates multi-dimensional immune information from flow cytometry, cytokine profiling, and immune checkpoint molecule assessments. The proposed model offers a new way to assess immune status and could serve as a valuable tool for clinical personalized treatment and prognostic evaluation.

## 1 Introduction

The tumor immune microenvironment (TME) is an intricate and dynamic network consisting of various cell types, signaling molecules, and cellular interactions (1). Unlike normal tissue, the TME is characterized by a diverse and evolving composition that can drastically influence tumor progression, immune surveillance, and therapeutic outcomes. The complexity of the TME arises from the following factors:

### 1.1 Cellular heterogeneity

The TME contains a broad spectrum of immune and non-immune cells, each playing distinct roles in either supporting or inhibiting tumor progression. Immune cells in the TME include cytotoxic T lymphocytes (CTLs), regulatory T cells (Tregs), B cells, natural killer (NK) cells, macrophages, and dendritic cells (DCs) (2). These immune cells do not only interact with tumor cells but also with other non-immune stromal cells like fibroblasts, endothelial cells, and the extracellular matrix, creating a highly heterogeneous and constantly changing microenvironment. The functional state and activation of these immune cells are influenced by various molecular signals and environmental factors within the TME (3).

### 1.2 Molecular signaling networks

In the TME, immune cells are constantly exposed to tumor-secreted factors, such as cytokines, chemokines, and growth factors, which profoundly shape their behavior. Tumor cells often secrete immunosuppressive cytokines like TGF-β and IL-10, which promote immune tolerance and inhibit anti-tumor immunity. On the other hand, pro-inflammatory cytokines such as IL-2, IFN-γ, and TNF-α are essential for activating immune responses. The balance between these pro-inflammatory and immunosuppressive signals plays a critical role in determining whether the immune response in the TME will lead to tumor control or immune evasion (4).

### 1.3 Immune evasion mechanisms

Tumor cells are adept at modulating the immune microenvironment to escape immune detection. This occurs through various mechanisms, including immune checkpoint activation (e.g., PD-1/PD-L1, CTLA-4, LAG-3) and the recruitment of Tregs and myeloid-derived suppressor cells (MDSCs), which inhibit immune activation. These immune evasion strategies are particularly evident in the case of immune checkpoint inhibitors (ICIs), which have shown promising therapeutic potential in certain cancers but often encounter resistance due to complex immune suppression in the TME. Therefore, understanding the multi-layered immune interactions within the TME is essential for developing more effective and personalized therapeutic approaches (5).

### 1.4 Tumor-stroma interaction

Beyond immune cells, the TME is influenced by various non-immune stromal cells, including fibroblasts, endothelial cells, and extracellular matrix components, which all contribute to the immune and tumor microenvironment. Cancer-associated fibroblasts (CAFs), for instance, secrete factors that modify immune cell behavior and tumor cell survival, while endothelial cells promote angiogenesis to supply the growing tumor with nutrients. These stromal components can also help in immune evasion by physically and chemically shielding tumor cells from immune cells or by recruiting immunosuppressive cells to the TME.

### 1.5 Spatio-temporal dynamics of the TME

The TME is not static but evolves dynamically over time, especially in response to therapeutic interventions. Spatial heterogeneity within the tumor tissue adds another layer of complexity, as different areas of the tumor may exhibit varying immune cell infiltrates and molecular signals. The central tumor region may be poorly oxygenated, leading to immune suppression, while the periphery of the tumor may exhibit higher levels of immune cell activity. Understanding these spatial and temporal changes is crucial for designing therapies that can target the immune system at multiple levels, from the cellular to the molecular.

## 2 Tumor local immune profiling challenges and peripheral blood immune profiling advantages

While intratumoral immune profiling offers the most direct assessment of local immune status, it faces significant technical and operational challenges:

- Sampling difficulties: Solid tumor biopsies often provide minimal tissue samples and may not represent the entire tumor's immune environment.
- Spatial heterogeneity: Different regions within a tumor may have significantly different immune profiles, and localized profiling may not reflect the overall immune landscape of the tumor.
- Technical limitations: Existing technologies, such as tissue-based cytometry techniques and immunohistochemistry, fail to comprehensively and accurately assess immune cell functions and interactions.

In contrast, peripheral blood immune profiling offers the following advantages:

- Ease of sampling: Blood collection is non-invasive, and it can be repeated multiple times, providing an opportunity for longitudinal monitoring.
- High reproducibility: Peripheral blood sampling allows for dynamic immune monitoring, enabling real-time tracking of changes in the immune system over the course of treatment.
- Operational practicality: Technologies like flow cytometry and cytokine profiling can accurately assess immune cell types, subtypes, and their functional states in peripheral blood, providing valuable clinical insights.

Studies have shown that immune cell populations in peripheral blood (such as T cells, Tregs, and B cells) correlate with responses to immunotherapy (6–9). Especially in situations where intratumoral immune profiling is limited, peripheral blood immune profiling provides an important complement for evaluating therapeutic efficacy and prognostic outcomes (10).

## 3 Immune structural model: inspired by protein structural hierarchy

**Figure 1:**
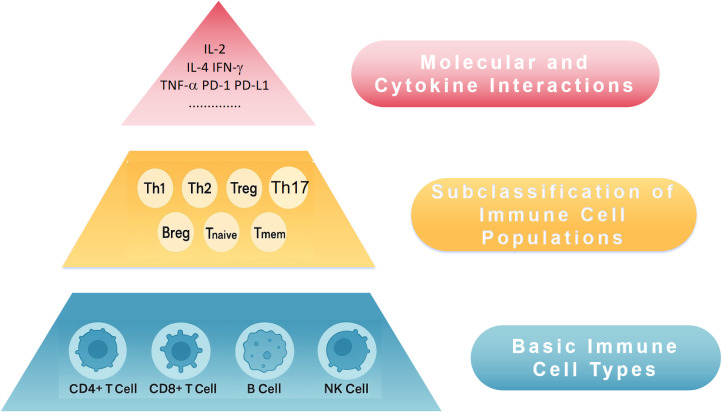
Conceptual three-tier immune structural model for peripheral blood immune profiling and ISS. ISS, Immune Scoring System; NK cell, natural killer cell; Th, T helper cell; Treg, regulatory T cell; Breg, regulatory B cell; IL-2, interleukin 2; IL-4, interleukin 4; IFN-γ, interferon-γ; TNF-α, tumor necrosis factor-α; PD-1, programmed death 1; PD-L1, programmed death ligand 1.

Proteins possess a primary structure (amino acid sequence), a secondary structure (α-helix, β-sheet), and a tertiary structure (3D conformation of molecules), which dictates their function through hierarchical organization (11). Drawing inspiration from this, we propose that immune information can also be stratified into three hierarchical levels as shown in **Figure 1**:

### 3.1 Primary immune structure — basic immune cell types

Peripheral blood analysis can assess basic immune cell populations, such as CD4+ T cells and CD8+ T cells, which are central to anti-tumor immunity. The dynamic balance between these two cell types is essential for overall immune function. Their proportions and numbers are fundamental indicators of immune status. Additionally, B cells and NK cells can be included as Primary indicators.

### 3.2 Secondary immune structure — subclassification of immune cell populations

Within the primary immune compartments, further categorization of immune cells into functional subsets provides deeper insights into immune function:

- CD4+ T cell subsets: Th1, Th2, Th17, Treg, etc.
- CD8+ T cell subsets: Activated T cells (e.g., CD69+), memory T cells (CD45RO+), naïve T cells (CD45RA+).
- B cells and NK cells: Further subclassification into memory, plasma, and cytotoxic/activated types.

This level of analysis provides a clearer understanding of how immune cells contribute to effective anti-tumor responses.

### 3.3 Tertiary immune structure — molecular and cytokine interactions beyond cellular composition, immune function is regulated by molecular interactions and cytokine networks

- Immune checkpoint molecules: PD-1, CTLA-4, LAG-3, etc.
- Cytokine environment: Pro-inflammatory cytokines (e.g., IL-2, IFN-γ), immunosuppressive cytokines (e.g., IL-10, TGF-β).
- Immune proteomics: Profiling peripheral blood proteins for immune activation or suppression markers.

The integration of these three levels (primary, secondary, tertiary) forms a comprehensive approach to quantifying immune function and provides a systematic method for immune scoring.

## 4 Constructing the peripheral blood immune scoring system

Based on the proposed immune structural model, we suggest constructing a multi-level, weighted immune scoring system:

ISS=w1×(Primary Score)+w2×(Secondary Score)+w3×(Tertiary Score).

- Primary Score (PS): Based on the proportions or numbers of CD4+ / CD8+ T cells, reflecting the foundation of immune status.
- Secondary Score (SS): Derived from the fine sub-populations of CD4+ and CD8+ T cells, including functional differentiation (e.g., Th1, Th2, Treg), activation (e.g., CD69+), and memory T cells (CD45RO+).
- Tertiary Score (TS): Quantifies the expression levels of immune checkpoint molecules (e.g., PD-1, LAG-3) and cytokines (e.g., IL-2, IFN-γ, IL-10).

### 4.1 Weighting the scores

1. Primary Structure is the most fundamental and should carry the largest weight, as it represents the basic immune equilibrium between major immune cell types (e.g., T cells). We recommend assigning a higher weight to this level, e.g., w1 = 0.5.
2. Secondary Structure provides more detailed functional information and is important for evaluating immune activation status. The weight for this level could be moderate, e.g., w2 = 0.3.
3. Tertiary Structure provides valuable molecular insights but is supplementary to the cellular-based scores. This level should have a smaller weight, e.g., w3 = 0.2.

**Figure 2:**
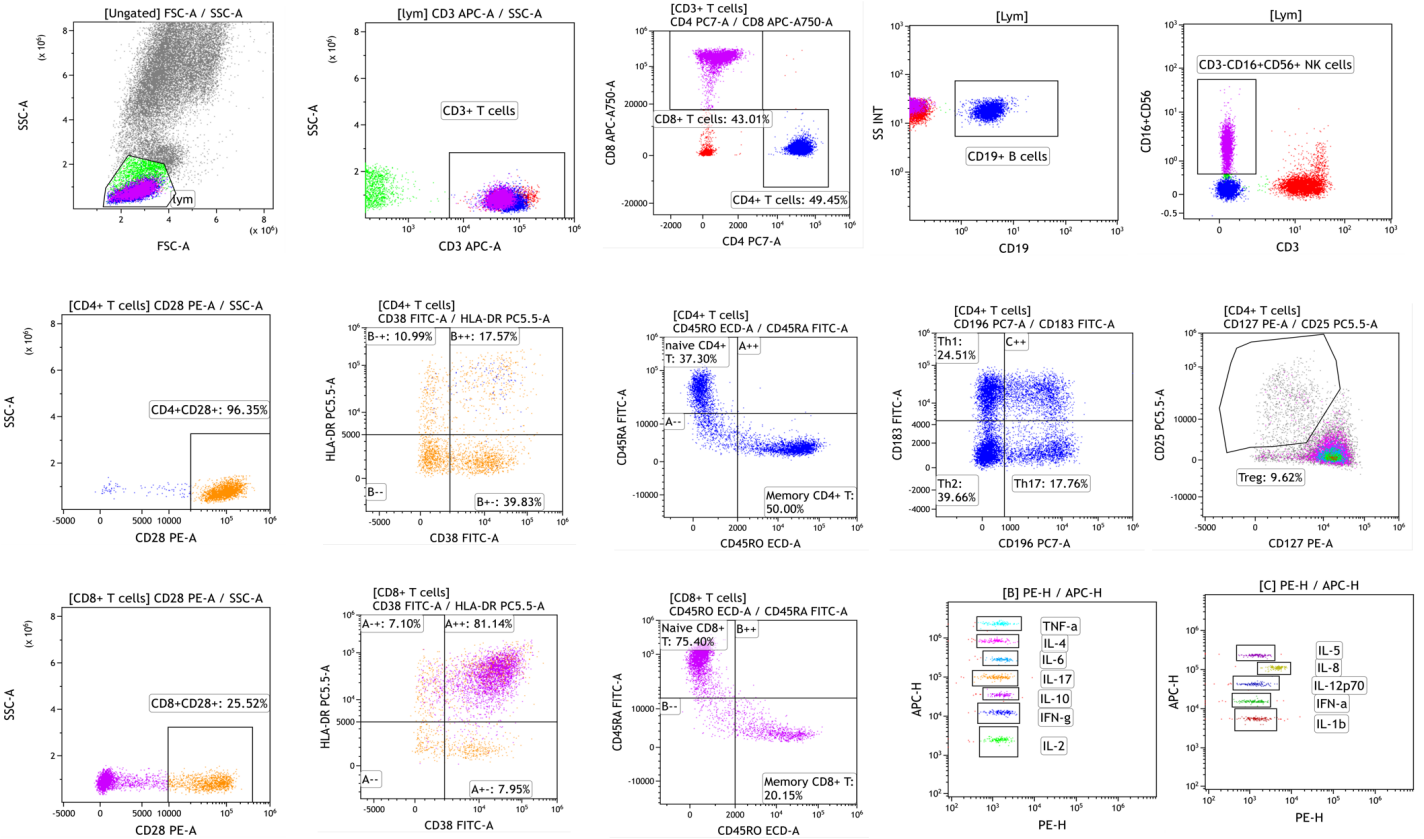
The workflow and schematic diagram of flow cytometric analysis of immune cell subsets and cytokine detection.

These weightings should be further optimized through clinical validation studies, where the optimal coefficients can be determined based on large-scale data. The methods and platforms are shown in **Table 1**. The workflow and schematic diagram of flow cytometric analysis of immune cell subsets and cytokine detection are shown in **Figure 2**.

**Table 1:** Candidate features for the ISS: definitions, measurement platforms, and expected directionality.

Tier	Feature (marker examples)	Measurement platform	Unit / readout	Expected directionality (example)
Primary	CD3+, CD4+, CD8+ T cell counts; CD4:CD8 ratio; CD19+ B cells; CD56+ NK cells	CBC; flow cytometry	cells/µL; % of lymphocytes	Low lymphocyte or high neutrophil/platelet counts → adverse prognosis
Secondary	CD4+ Treg (CD25+FoxP3+ or CD25+CD127^low); Th1/Th2/Th17 subsets; CD8+ memory/naïve (CD45RO/CD45RA); activation (CD69, HLA-DR, CD38); Breg (CD19+CD24^hiCD38^hi)	Multicolor flow cytometry; intracellular cytokine staining	% of parent subset; MFI (marker expression)	High Treg% → immunosuppression; high activated CD8+% → favorable immune activation
Tertiary	Immune checkpoints (PD-1, PD-L1, CTLA-4, LAG-3, TIM-3); cytokines (IL-2, IFN-γ, TNF-α, IL-6, IL-10, TGF-β); serum LDH	Flow cytometry (surface); ELISA, Luminex, MSD multiplex assays; clinical chemistry	% positive cells; pg/mL; U/L (for LDH)	Elevated IL-10/TGF-β→ immunosuppression; elevated IFN-γ/IL-2 → immune activation
Other candidates	Myeloid-derived suppressor cells (CD11b+CD33+HLA-DR^low); CRP; ESR	Flow cytometry; clinical chemistry	cells/µL; mg/L	High MDSCs or elevated CRP/ESR → systemic immunosuppression

ISS, Immune Scoring System; CBC, Complete blood count; Treg, regulatory T cell; Foxp3, forkhead box P3; Th, T helper cell; HLA-DR, human leukocyte antigen DR; Breg, regulatory B cell; MFI, mean fluorescence intensity; PD-1, programmed death 1; PD-L1, programmed death ligand 1; CTLA-4, cytotoxic T lymphocyte associated protein 4; LAG-3, lymphocyte activation gene 3; Tim-3, T cell immunoglobulin and mucin domain containing 3; IL-2, interleukin 2; IFN-γ, interferon-γ; TNF-α, tumor necrosis factor-α; IL-6, interleukin 6; IL-10, interleukin 10; TGF-β, transforming growth factor-β; LDH, lactate dehydrogenase; ELISA, enzyme linked immunosorbent assay; MSD, meso scale discovery; CRP, c-reactive protein; ESR, erythrocyte sedimentation rate; MDSCs, myeloid derived suppressor cells;

## 5 Clinical and research applications

### 5.1 Guiding immunotherapy decisions

The ISS can help predict which patients will respond best to immune checkpoint inhibitors (PD-1/PD-L1, CTLA-4, etc.) or other immunotherapies.

### 5.2 Longitudinal immune monitoring

Tracking the ISS over time can provide insights into immune dynamics during treatment, offering a tool for assessing immune response and resistance.

### 5.3 Prognostic and risk stratification

The ISS could serve as a biomarker for predicting cancer progression, survival rates, and overall prognosis, helping clinicians manage treatment strategies more effectively.

### 5.4 Enhancing immunobiology research

The ISS offers a standardized way to integrate peripheral blood immune data and allows for comparative studies across different research groups, fostering a better understanding of tumor immune environments.

## 6 Innovations and limitations

### 6.1 Innovations

- The immune structural model is the first to apply a three-tiered system to classify immune status in peripheral blood, based on functional immune cells, their subtypes, and molecular interactions.
- This model facilitates detailed yet practical immune assessment using routine clinical blood samples.

### 6.2 Limitations

- The model requires validation through large-scale clinical studies to establish the most reliable biomarkers and weightings for scoring.
- While peripheral blood offers valuable insights, it may not fully replicate the local immune status within the tumor microenvironment, and the ISS should be used in conjunction with other clinical and pathological data.

## 7 Discussion

### 7.1 Relationship to established blood-based indices

Several simple blood-derived indices have been widely studied as prognostic or predictive biomarkers in oncology, including the neutrophil-to-lymphocyte ratio (NLR), derived NLR (dNLR), platelet-to-lymphocyte ratio (PLR), systemic immune-inflammation index (SII), and the lung immune prognostic index (LIPI) (12). These indices are attractive because they can be calculated from routine complete blood counts and have been associated with outcomes across multiple tumor types. However, they capture only gross features of systemic inflammation or myeloid/lymphoid balance and do not directly represent immune functional states, cellular subsets, or molecular checkpoint activity. By design, the ISS proposed here complements these indices by integrating three hierarchical layers of immune information (basic immune cell populations, functional/phenotypic subsets, and molecular/cytokine signals) into a composite metric that aims to better reflect immune competence and treatment-relevant biology, as shown in **Table 2**. Where simple indices (e.g., NLR, SII, LIPI) provide rapid screening and risk stratification, ISS is intended to provide greater mechanistic resolution and clinical granularity, particularly for immunotherapy decision-making and longitudinal immune monitoring.

**Table 2:** Comparison of existing blood-based indices versus the proposed ISS.

Score	Formula / components	Typical use	Strengths	Limitations	How ISS complements
NLR	Neutrophils / Lymphocytes	Prognosis across cancers	Simple, inexpensive, routinely available	Non-specific; reflects inflammation but not functional immune status	ISS incorporates lymphocyte subsets, functional differentiation, and molecular checkpoints
PLR	Platelets / Lymphocytes	Prognosis, thrombosis-related risk	Simple, available from CBC	Similar limitations as NLR; does not capture immune activation states	ISS adds immune phenotype and cytokine context
SII	(Platelets × Neutrophils) / Lymphocytes	Prognostic biomarker in multiple cancers	Integrates three blood count parameters	Still reflects coarse inflammation rather than functional immunity	ISS provides multi-dimensional mechanistic resolution
dNLR	Neutrophils / (Leukocytes − Neutrophils)	Used in LIPI and ICI studies	Simple, validated in ICI cohorts	Limited scope; lacks molecular and subset detail	ISS provides checkpoint and cytokine-level information
LIPI	dNLR + LDH	Prognostic index in NSCLC patients on ICIs	Combines systemic inflammation and tumor burden	Specific to certain settings; limited mechanistic insight	ISS is generalizable, mechanistic, and can be applied across tumor types
ISS (proposed)	Weighted composite of Primary, Secondary, and Tertiary immune structures	Predictive/prognostic biomarker, immunotherapy decision-making, longitudinal monitoring	Multi-layered, mechanistically informed, adaptable	Requires validation; more resource-intensive than CBC	Complements existing indices by offering functional and molecular resolution

ISS, immune scoring system; NLR, neutrophil-to-lymphocyte ratio; PLR, platelet-to-lymphocyte ratio; CBC, complete blood count; SII, systemic immune-inflammation index; dNLR, derived neutrophil-to-lymphocyte ratio; LIPI, lung immune prognostic index; ICIs, immune checkpoint inhibitors; LDH, lactate dehydrogenase; NSCLC, non-small cell lung cancer;

### 7.2 Data-driven derivation of ISS weights and model development

The numeric weights used in the illustrative ISS (w1, w2, w3) are placeholders for conceptual demonstration. For clinical implementation, we propose a data-driven derivation and validation workflow. Candidate features will include primary counts (e.g., absolute CD4/CD8, CD4:CD8 ratio, NK and B cell counts), secondary phenotypes (e.g., %Treg, %memory CD8, activation markers), and tertiary measures (e.g., PD-1 expression, circulating cytokine concentrations). Feature selection and weight estimation should be performed in a derivation cohort using penalized regression approaches (e.g., LASSO or elastic net) or other regularized/statistical learning methods, with model tuning performed by nested cross-validation to avoid optimistic bias. Model performance should be evaluated using discrimination measures (AUC for binary outcomes; C-index for time-to-event outcomes), calibration plots, and decision curve analysis. Final weights and the scoring algorithm must be locked before testing in an independent validation cohort. Pre-specified clinical endpoints for optimization and validation might include overall survival (OS), progression-free survival (PFS), and objective response rate (ORR); time points and censoring rules should be pre-defined following standard reporting guidelines for prediction modeling (e.g., TRIPOD-style recommendations).

### 7.3 Pre-analytical and technical considerations

Peripheral immune profiling is sensitive to pre-analytical and analytical variation. Prior studies have shown that blood processing delays, tube type, temperature during transport, and processing protocols can materially affect both cytokine measurements and flow cytometric readouts. To improve reproducibility and to facilitate multi-center validation, we recommend defining and reporting minimal pre-analytical standards: (1) specify anticoagulant/tube type for each assay; (2) record time from venipuncture to processing and aim for processing within 2–4 hours when feasible; (3) standardize sample handling temperature (room temperature vs refrigerated) and centrifugation protocols for plasma/serum; (4) use standardized panels with published gating strategies and include internal controls; and (5) include replicate measures or internal reference samples for longitudinal studies. Adopting and reporting these standards will reduce methodological heterogeneity and improve comparability between cohorts.

### 7.4 Clinical covariates and confounder control

Because peripheral immune measures are affected by clinical status and concurrent medications, analytical models assessing ISS performance should adjust for plausible confounders. At minimum, we recommend collecting and adjusting for: age, sex, tumor type and stage, tumor burden (radiographic or measurable disease), ECOG performance status, baseline systemic inflammation markers (CRP, ESR), recent infections, chemotherapy/immunotherapy history, and concurrent medications known to affect immune parameters (systemic corticosteroids, proton pump inhibitors, immunosuppressants). Statistical approaches should include multivariable regression (Cox proportional hazards for time-to-event endpoints) and sensitivity analyses stratified by key factors (e.g., steroid use yes/no). Propensity-score adjustment or inverse probability weighting may be considered in observational cohorts where treatment allocation or supportive medications differ between groups.

### 7.5 Validation strategy and proposed clinical use cases

To establish clinical utility, ISS development should follow a two-stage strategy: (1) derivation (training) phase and (2) independent validation phase. In the derivation phase, candidate features and weights will be estimated using one or more well-annotated cohorts with pre-specified clinical endpoints (e.g., OS, PFS, ORR). Internal validation methods (cross-validation, bootstrap) will be used during model building to limit overfitting. The finalized scoring algorithm must then be tested in at least one external validation cohort from a separate institution or clinical trial to assess generalizability; performance metrics should include discrimination (C-index, AUC), calibration (calibration slope and plots), and clinical net benefit (decision curve analysis). For dynamic monitoring, time-dependent ROC analyses and joint models or landmark analyses can be used to quantify how longitudinal changes in ISS relate to subsequent outcomes.

Potential clinical use cases to test in validation studies include:

- Baseline ISS as a predictive biomarker to enrich or stratify patients for immune checkpoint inhibitors.
- Early on-treatment ISS change as an indicator of response vs. resistance (to guide continuation vs. switch of therapy).
- Longitudinal ISS trajectories for relapse surveillance after curative-intent therapy.

Sample size considerations and number-of-events rules should follow standard practice for prediction models (e.g., ensuring adequate events per variable during derivation). External validation is essential for assessing reproducibility and transportability prior to clinical implementation.

## 8 Conclusion

The proposed immune structural model provides a novel framework for understanding and quantifying immune function in cancer patients using peripheral blood. By integrating immune cell composition, functional subtypes, and molecular signals, this model facilitates the development of a comprehensive ISS, which could significantly impact clinical decision-making for cancer immunotherapy and precision medicine. Future research should focus on validating this scoring system across various cancer types and integrating it into clinical practice.

## Data Availability

The raw data supporting the conclusions of this article will be made available by the authors, without undue reservation.
